# Metastatic Melanoma Causing Intussusception in the Small Bowel: A Case Report

**DOI:** 10.7759/cureus.95510

**Published:** 2025-10-27

**Authors:** Sonakshi Nemchand, Ling Fung Chan, Sivakumaran Sabanathan

**Affiliations:** 1 General Surgery, Wrexham Maelor Hospital, Wrexham, GBR

**Keywords:** hmb45 melanocytic marker, ileal intussusception, jejunal intussusception, melanoma, neoplastic cells, primary malignant melanoma, small bowel intussusception, sox10 melanocytic marker

## Abstract

Intussusception is a condition in which a part of the intestine telescopes into an adjacent segment of the bowel. This can obstruct the passage of food and fluids and may compromise the blood supply, leading to bowel obstruction, ischemia, infection, or perforation. Common symptoms include constipation, bloating, nausea, vomiting, and abdominal pain. Diagnosis is typically confirmed with computed tomography (CT) or ultrasonography, and definitive treatment involves bowel resection or reduction. We present the case of a 54-year-old man with a history of metastatic malignant melanoma, previously in complete remission following immunotherapy, who developed small bowel obstruction secondary to intussusception. He initially presented with a three-week history of loose stools, followed by a week of bilious vomiting, abdominal pain, and absence of bowel movements. A CT scan of the abdomen and pelvis revealed two sites of intussusception at the distal jejunum and distal ileum. The patient underwent exploratory laparotomy with resection of both affected small bowel segments and primary anastomoses. Postoperatively, he made a full recovery without complications. Histopathology confirmed metastatic malignant melanoma infiltrating both bowel segments, with clear resection margins and no lymphovascular invasion. Melanoma most commonly metastasizes to the gastrointestinal tract via hematogenous or lymphatic spread, with the small bowel being the most frequent site of involvement. This case illustrates a rare yet clinically significant instance of metastatic melanoma presenting as dual-site small bowel intussusception occurring more than a year after apparent remission with immunotherapy. It emphasizes that gastrointestinal metastases from melanoma can develop insidiously, even in the absence of cutaneous recurrence, and may present solely with nonspecific obstructive symptoms. CT remains the cornerstone of diagnosis, while surgical resection serves as both diagnostic and curative. Importantly, this case underscores the need for ongoing vigilance and long-term follow-up in patients with a history of metastatic melanoma, as timely recognition and surgical management can significantly improve outcomes and quality of life.

## Introduction

Intussusception is a condition in which part of the intestine telescopes into an adjacent section of the bowel. This can make it difficult for food and fluids to pass through the affected segment, leading to bowel obstruction. Additionally, intussusception may cut off the blood supply to the involved section of the intestine, leading to ischemia, infection, and in severe cases, perforation with peritonitis [1]. 

Symptoms of intussusception include constipation, bloating, nausea, vomiting, and abdominal pain. In adults, the diagnosis typically involves imaging studies such as computed tomography (CT) or ultrasonography [1]. A barium enema may also be used when colonic or ileo-colic intussusception is suspected [2]. Treatment options include surgical intervention, such as resection or reduction, which is considered definitive [3]. However, conservative management with analgesics and antiemetics may be appropriate for patients who are not suitable candidates for surgery [4].

Epidemiologically, intussusception is more common in children and is considered extremely rare in adults. The rate of abdominal surgeries due to intussusception is approximately one in 20 for children, compared to one in 1,300 for adults [1]. In adults, intussusception can be classified by location, either in the small or large bowel. The most common cause in the large bowel is malignancy (60%), whereas in the small bowel, benign tumors are responsible for approximately 60% of cases. In contrast, only about 30% of small bowel intussusceptions are due to malignancy [5]. Among malignant tumors in the gastrointestinal (GI) tract, malignant melanoma accounts for just 1-3% [6], and most of these cases are secondary, originating from sites such as the skin. 

## Case presentation

A 54-year-old man presented with a three-week history of loose stool. In the week prior to presentation, he developed bilious vomiting, was unable to tolerate oral intake, and had not opened his bowels for seven days. He was also complaining of significant abdominal pain. He had a history of metastatic melanoma (stage IVb) on the skin with a complete response to immunotherapy (completed September 2023). His relevant past medical history included stage 1a malignant melanoma on the skin of the left upper chest in 2010. He also had stage 1b malignant melanoma on the skin of the right upper back, with a Breslow thickness of 0.8 mm, which was excised in 2014.

**Figure 1:**
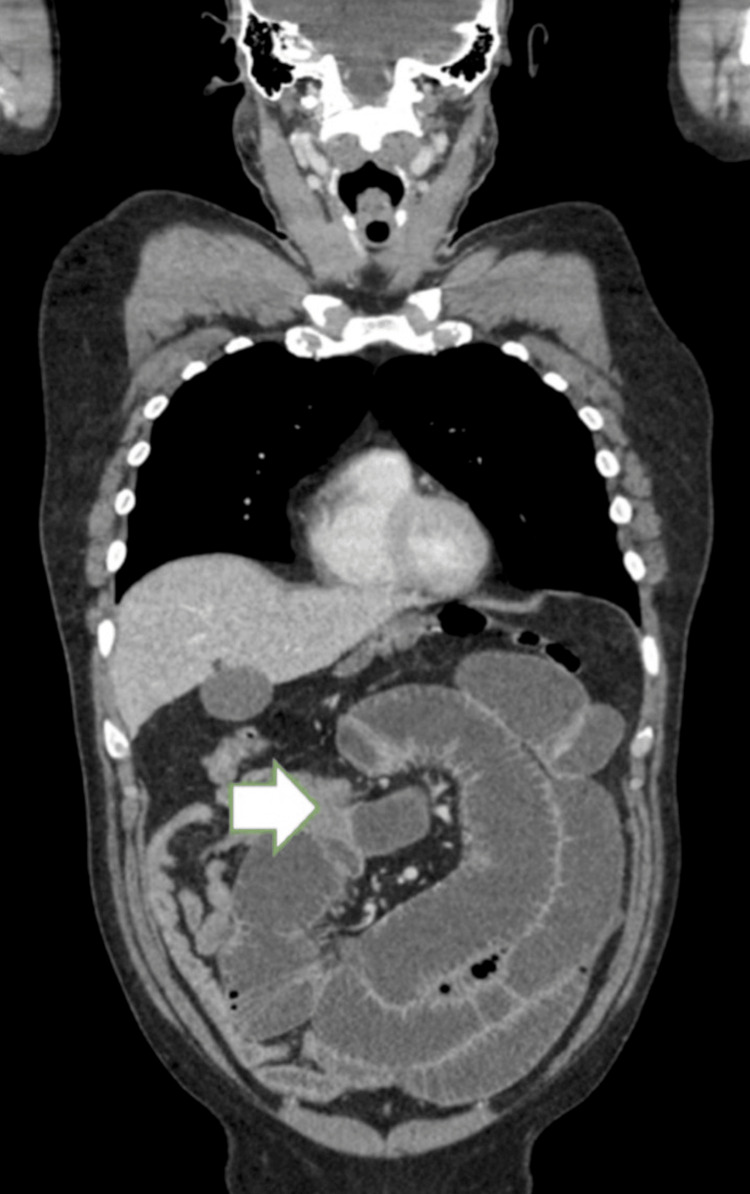
Contrast-enhanced CT abdomen and pelvis (coronal section) showing distal jejunal obstruction (white arrow)

**Figure 2:**
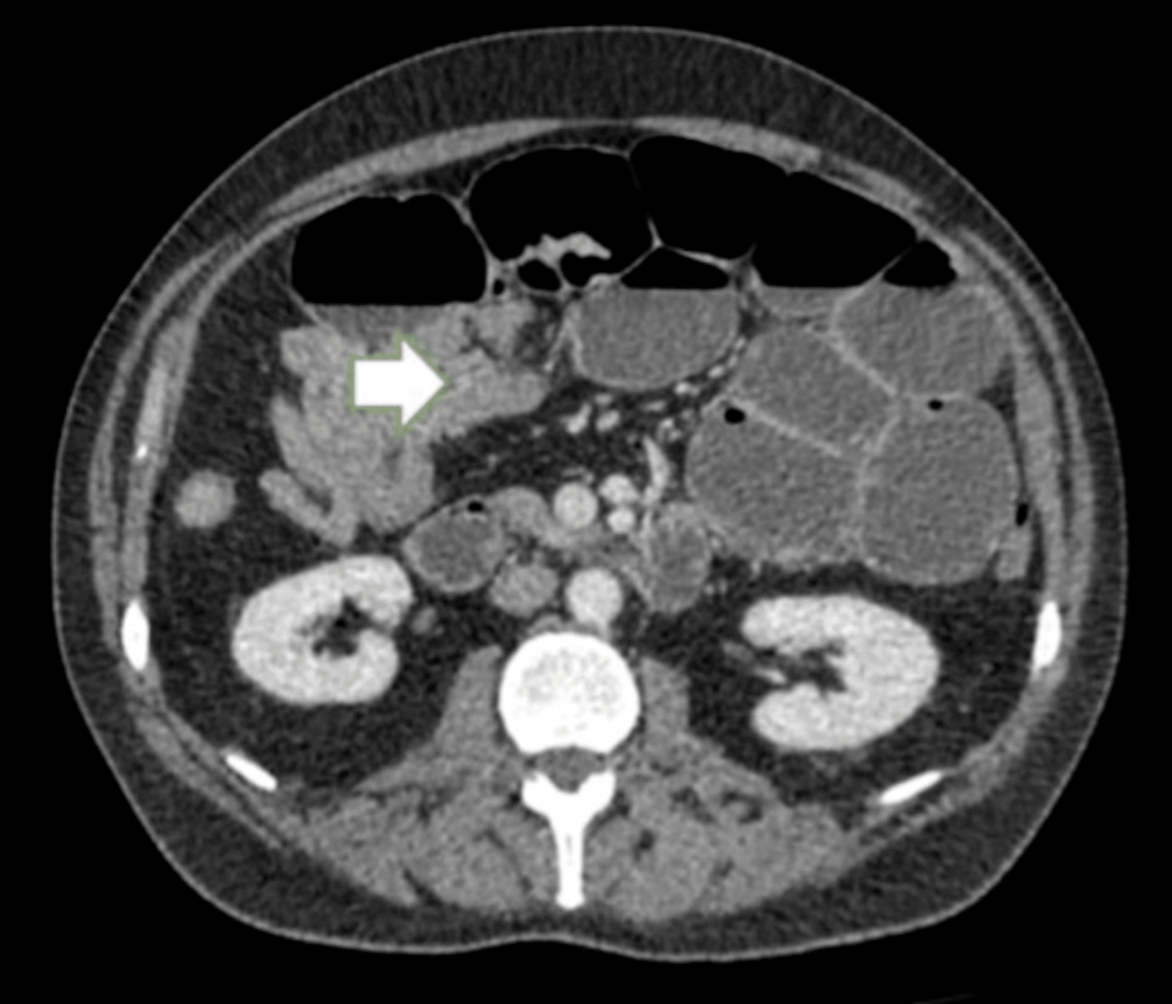
Contrast-enhanced CT abdomen and pelvis (axial section) showing jejunal obstruction (white arrow)

**Figure 3:**
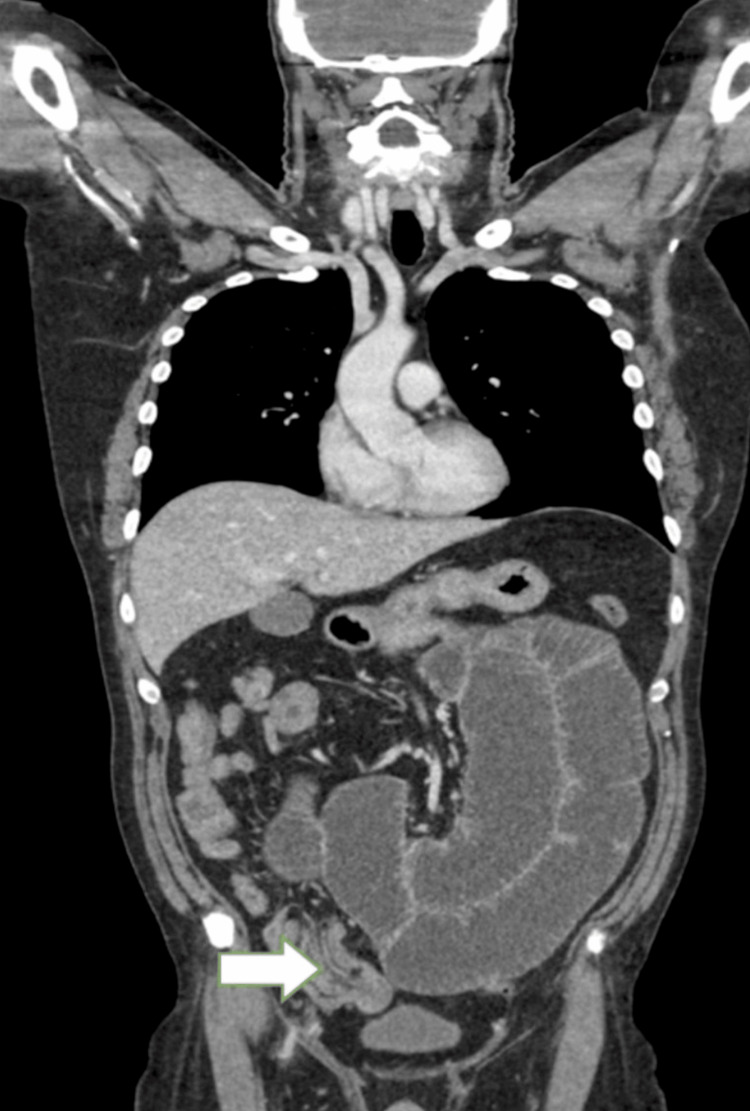
Contrast-enhanced CT abdomen and pelvis (coronal section) showing distal ileum obstruction (white arrow)

In light of this, a contrast-enhanced CT abdomen and pelvis (CTAP) was performed in February 2025, which demonstrated significant small bowel obstruction with a transitional point in the distal jejunum caused by intussuception (Figures 1 and 2) and a further large intussusception in the distal ileum (Figures 2, 3).

**Figure 4:**
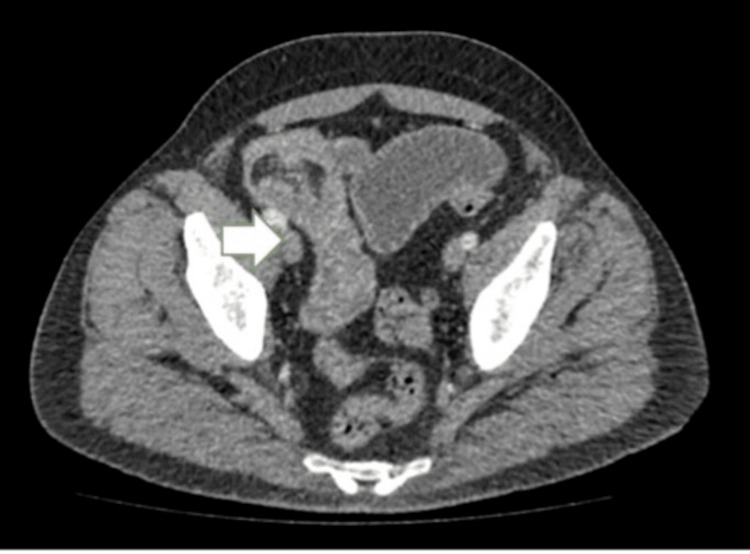
Contrast-enhanced CT abdomen and pelvis (axial section) showing ileum obstruction (white arrow)

IV fluids were commenced, and a Ryle's tube was inserted. Given this, the patient consented to and underwent an exploratory laparotomy with small bowel resection and two primary anastomoses, thus avoiding the need for stoma formation. He remained in the intensive care unit briefly for blood pressure support as he could not wean off vasopressor; however, he recovered well following surgery, and he was de-escalated to the surgical ward after he was off vasopressor and his blood pressure was stable. A follow-up appointment was scheduled based on his histology results. For his melanoma on his skin, he is due for follow-up in the Dermatology Department in six months.

Histopathology

**Figure 5:**
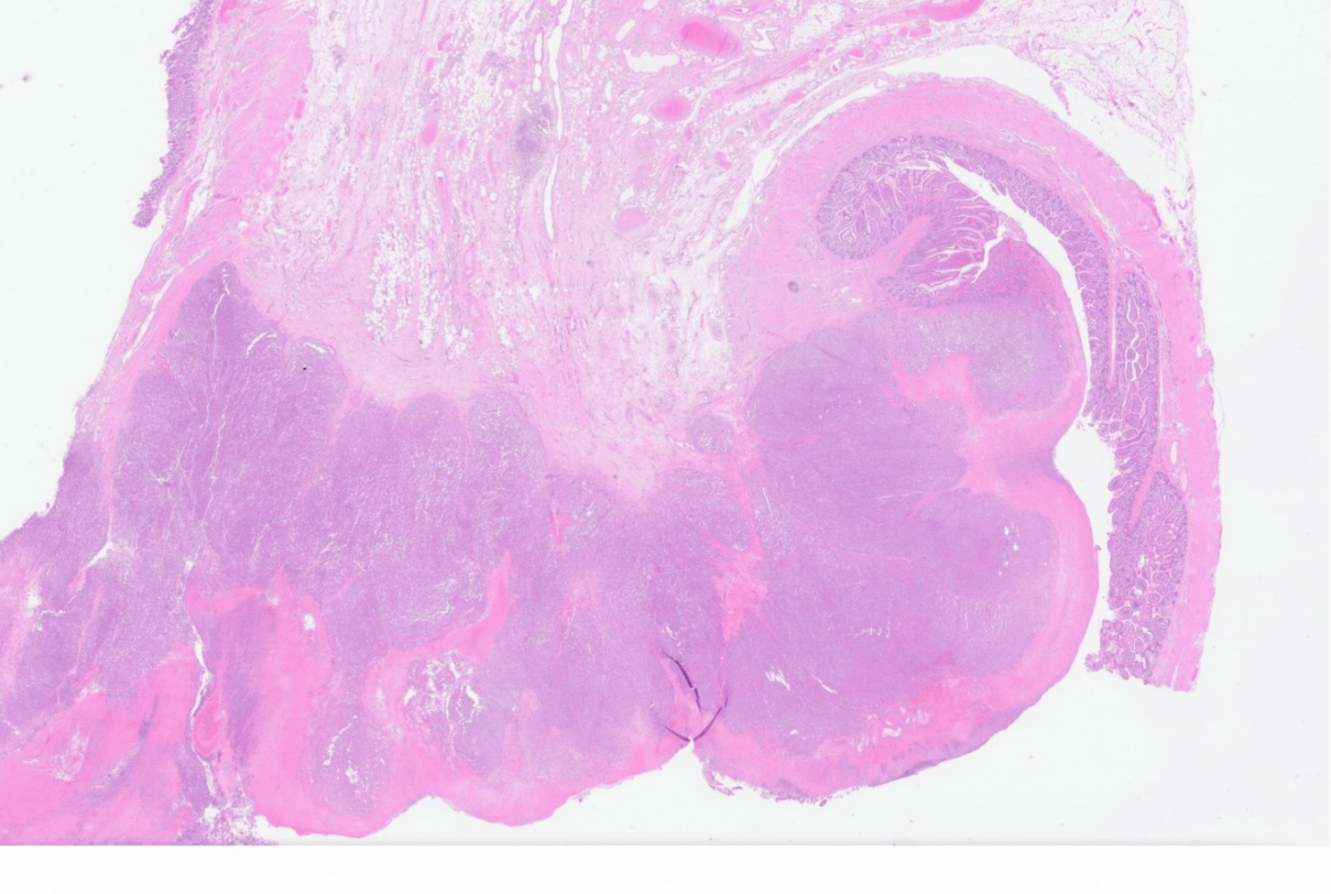
Sheets of poorly differentiated cells infiltrating mucosa & submucosa of small bowel

On gross examination, two polypoid lesions obstructing the lumina of both segments from the distal jejunum and the distal ileum were identified. Histologic examination revealed a malignant neoplasm infiltrating into the submucosa and muscularis propria, composed of sheets of epithelioid neoplastic cells with enlarged, irregular nuclei and prominent nucleoli on H&E stain. The background small bowel mucosa is normal and free of dysplasia (Figure 5).

**Figure 6:**
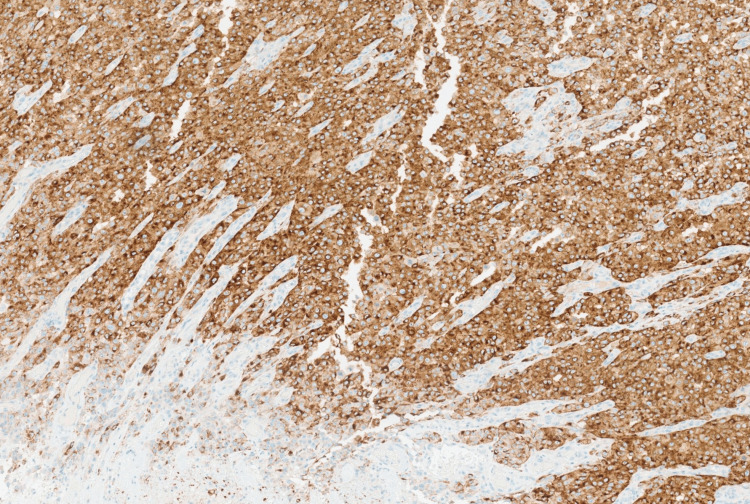
Neoplastic cells are positive for HMB45 melanocytic marker

**Figure 7:**
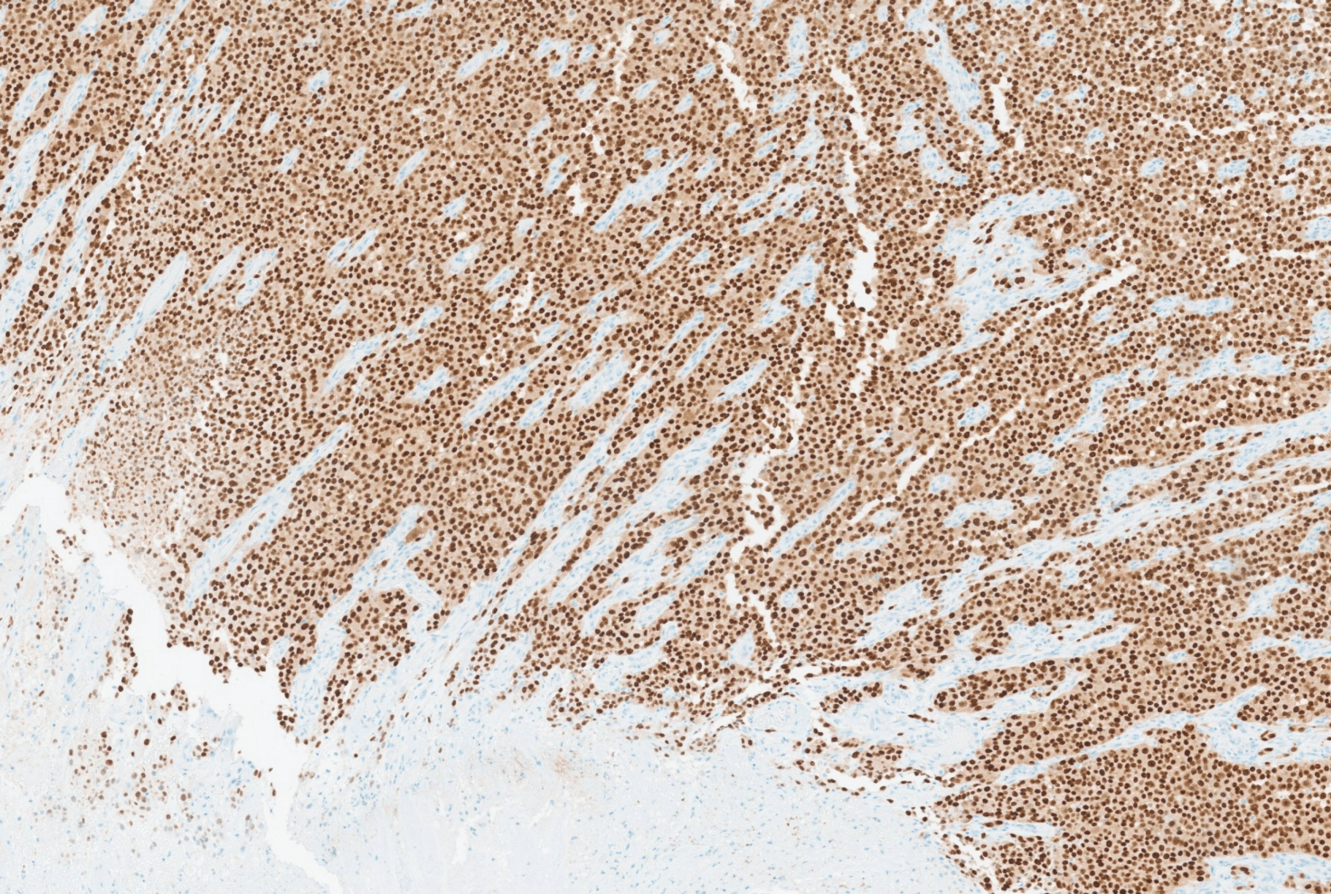
Neoplastic cells are positive for SOX10 melanocytic marker

**Figure 8:**
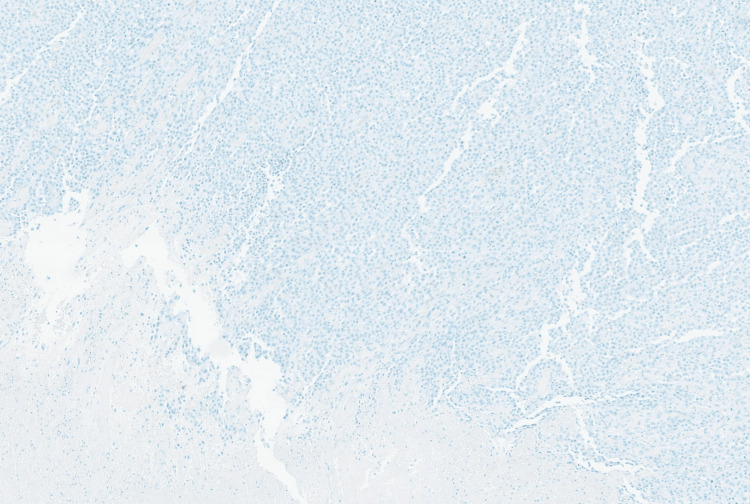
Neoplastic cells are negative for MNF116 epithelial marker

**Figure 9:**
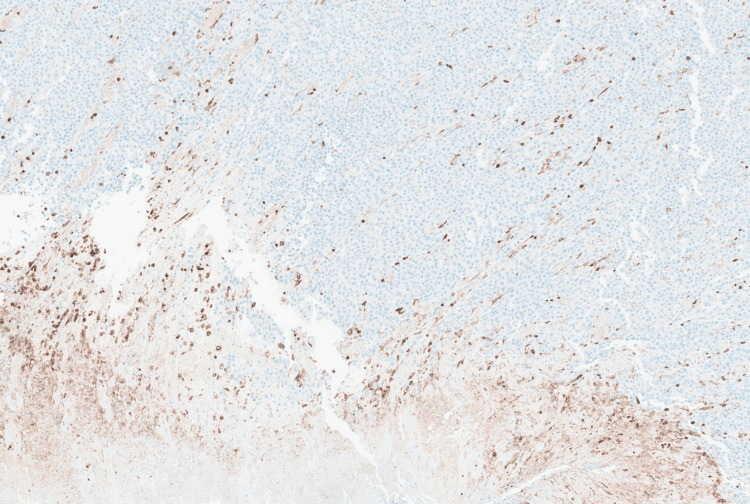
Neoplastic cells are negative for CD 45 pan leukocyte marker

Immunohistochemical stains revealed strong positivity for HMB45 (Figure 6) and SOX10 (Figure 7), while negative for the epithelial marker MNF116 (Figure 8) and leukocytic marker CD45 (Figure 9), confirming melanocytic origin.

## Discussion

Intussusception in adults is a rare clinical entity, accounting for only 1-5% of cases of intestinal obstruction, and unlike paediatric intussusception, it is almost always associated with an underlying pathological lead point, frequently a tumour [7]. In the small bowel, intussusception is more commonly caused by benign lesions, such as lipomas, polyps, or Meckel’s diverticulum; however, approximately 30% of small bowel cases are due to malignancy, particularly metastatic disease [5].

Malignant melanoma is known for its unpredictable metastatic pattern and ability to spread to nearly any organ, including the GI tract. While GI metastases from melanoma are often found at autopsy, reported in up to 60% of cases, they are clinically detected in only about 1-5% of patients during their lifetime [7,8]. The small intestine is the most frequent site of GI involvement due to its rich vascular supply, and metastases may remain asymptomatic or present with non-specific symptoms such as abdominal pain, anaemia, or obstruction [9]. Although malignant melanoma is rare in the GI tract, it is the most common cancer to metastasize to the small bowel, accounting for 51-71% of cases [10]. This may be related to the high expression of C-C motif chemokine ligand 25 (CCL25), a ligand for the C-C chemokine receptor type 9 (CCR9), in the small intestine, which may facilitate migration of melanoma cells from the skin to the small bowel [11]. Once present, melanoma in the small bowel can act as a lead point; during bowel contractions, this disrupts normal peristalsis and may result in telescoping of the bowel. In the present case, the patient developed two sites of small bowel intussusception secondary to metastatic melanoma, manifesting as small bowel obstruction. Such presentations are uncommon but have been reported in the literature and highlight the diagnostic challenge in patients with a remote history of melanoma [12].

CT imaging plays a crucial role in identifying intussusception in adults, with characteristic findings including a “target” or “sausage-shaped” soft tissue mass with mesenteric fat and vessels [13]. In this patient, cross-sectional imaging accurately identified two distinct sites of intussusception, prompting surgical intervention.

Surgical resection remains the mainstay of treatment in adult intussusception, particularly when there is a concern for malignancy. Reduction of the intussuscepted segment prior to resection is controversial due to the risk of intraluminal tumour cell dissemination or venous embolization [14]. In this case, en bloc resection with primary anastomosis was performed, thereby avoiding stoma formation and resulting in favourable postoperative recovery.

Histopathology confirmed metastatic melanoma as the lead point in both resected segments, with clear margins and no lymphovascular invasion, consistent with previous reports showing that surgical resection can be both diagnostic and therapeutic [15]. Despite the patient’s prior complete response to immunotherapy, this case underscores the potential for late or isolated metastatic recurrence of melanoma within the GI tract [8,10].

Small-bowel evaluation modalities include capsule endoscopy (CE), barium studies, and CT. In comparative series, CE has shown higher sensitivity for small-bowel tumours (~79.6%) than barium (43.9%) or CT (40.4%) [16]. In our patient, CE was not pursued, given the obstructive presentation. Earlier-stage disease is associated with lower mortality; in a Surveillance, Epidemiology, and End Results (SEER)-based analysis, Grade I had a lower hazard of death than Grade IV (HR 0.535 vs 1.108) [17].

## Conclusions

Adult intussusception remains an uncommon but important cause of small bowel obstruction, and metastatic melanoma should be recognized as a potential underlying etiology, even years after apparent remission. This case highlights the diagnostic challenge posed by non-specific GI symptoms in patients with a history of melanoma and emphasizes the pivotal role of CT imaging in identifying intussusception. Definitive surgical resection remains the cornerstone of management, as it is not only therapeutic but also provides tissue for histological confirmation, which is critical for guiding further treatment. Although systemic therapies such as immunotherapy and targeted agents have transformed melanoma outcomes, there is currently no established role for neoadjuvant treatment in isolated GI metastases presenting with acute obstruction. In such settings, timely surgical intervention remains essential for both diagnosis and symptom resolution.

Given the high predilection of melanoma for the GI tract and its ability to present with isolated or late metastases, clinicians should maintain vigilance during follow-up. Incorporating appropriate surveillance strategies and considering advanced imaging modalities in selected high-risk patients may facilitate earlier detection of small bowel involvement. Ultimately, prompt recognition, timely intervention, and long-term multidisciplinary monitoring are key to optimizing outcomes in patients with metastatic melanoma presenting with intestinal complications.
